# Untying the Knot: A Fully Recyclable, Solvent‐Free, Wide‐Spectral Photocurable Thermoset Adhesive

**DOI:** 10.1002/adma.202502040

**Published:** 2025-05-16

**Authors:** Natanel Jarach, Michal Cohen, Rivka Gitt, Hanna Dodiuk, Samuel Kenig, Shlomo Magdassi

**Affiliations:** ^1^ The Department of Polymer Materials Engineering Pernick Faculty of Engineering Shenkar – Engineering. Design. Art Raman‐Gan 5252679 Israel; ^2^ Institute of Chemistry and Center for Nanoscience and Nanotechnology The Hebrew University of Jerusalem Jerusalem 91904 Israel

**Keywords:** adhesives, covalent adaptable networks, recycling, thermosets, vitrimers

## Abstract

Adhesives are vital in industries ranging from aerospace to consumer electronics. However, their reliance on non‐recyclable polymers makes them suitable for one‐time use and results in significant economic and environmental challenges. Reversible adhesives, based on covalent adaptable networks, address these issues and open possibilities for applications, including recyclable multi‐layer packaging, removable labels, and fully recyclable electronics – batteries, smartphones, etc. Despite progress in dynamic and reversible bonds, current solutions often compromise performance or the ability to detach from substrates. This study proposes reversible high‐performance adhesives that can undergo cycles of bonding‐debonding without commonly reported need to add solvent, high‐temperature processing or use deep‐UV irradiation while maintaining adhesives' functionality and reducing environmental impact. The bonding is done by rapid photocuring, which occurs within 30 seconds under irradiation at a wide range of visible wavelengths (400–650 nm) while achieving constant adhesion strength across diverse substrates, including underwater adhesion. De‐bonding is achieved using a simple household microwave. Furthermore, the adhesive's transparency and high refractive index enable its use in various optical applications, whereas its robustness in wet conditions expands its potential for bioadaptive or underwater systems. This adhesive represents a significant step toward sustainable adhesives that seamlessly integrate high performance with circular‐economy principles.

## Graphical abstract

This study introduces a solvent‐free, recyclable adhesive that cures rapidly under visible light (400–650 nm) while maintaining robust adhesion across diverse substrates. Recyclable via simple microwave irradiation, it combines high performance with optical and bioadaptive potential, representing a significant advancement toward sustainable, circular‐economy‐compatible adhesive technologies.

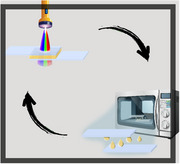

## 1 Introduction

From consumer electronics to aerospace, adhesives play an essential role in modern society. While the 2024 global adhesives market reached 92.6 billion dollars,^[^ ^1^ ^]^ or 122 billion dollars, including sealants,^[^ ^2^ ^]^ its reliance on irreversible and unrecyclable thermoset systems presents technical constraints, environmental and economic challenges, including material waste, and limitation of recycling processes.^[^ ^3^ ^]^

Addressing these challenges, researchers have explored the dynamic and reversible chemistry, known as covalent adaptable networks (CANs) or vitrimers, to develop debonding‐on‐demand and recyclable adhesives, focusing on thermal‐responsive bonds such as disulfides,^[^ ^4^, ^5^, ^6^, ^7^, ^8^ ^]^ β‐hydroxy esters,^[^ ^9^, ^10^, ^11^, ^12^ ^]^ or Diels‐Alder^[^ ^13^, ^14^ ^]^ or photo‐responsive bonds like azobenzene,^[^ ^15^, ^16^, ^17^, ^18^ ^]^ spiropyran,^[^ ^19^, ^20^ ^]^ or [2 + 2] cycloaddition reactions.^[^ ^21^, ^22^, ^23^, ^24^, ^25^, ^26^ ^]^ One example of such debondable adhesive has recently been introduced by Apple Inc. in their iPhone^®^ 16, facilitating simple battery removal in case of a phone malfunction.^[^ ^27^ ^]^ Current approaches for CANs‐based applications can be categorized into two: The first involves distinct chemistries for bonding and debonding, resulting in high‐performance adhesives, but often characterized by complex synthesis and reduced sustainability due to the adhesives' inability to completely dissociate from their substrates. The second approach utilizes a single bonding‐debonding mechanism that typically requires extreme conditions, such as elevated temperatures or specialized activation techniques. These two approaches highlight the need for solutions balancing sustainability with performance.

α‐Lipoic Acid (ALA), a naturally occurring cyclic disulfide, has shown promise in CANs using thermal,^[^ ^28^, ^29^, ^30^, ^31^, ^32^, ^33^, ^34^ ^]^ cationic,^[^ ^35^, ^36^ ^]^ or radical^[^ ^33^, ^37^, ^38^, ^39^, ^40^, ^41^, ^42^, ^43^ ^]^ ring‐opening polymerization. Despite their potential, current ALA‐based adhesives suffer some significant drawbacks,^[^ ^34^, ^36^, ^44^, ^45^, ^46^, ^47^, ^48^, ^49^ ^]^ including the need for solvents, high temperatures, or narrow‐spectrum UV light for photo‐polymerization. Moreover, they require high temperatures or adding solvents for recycling. For example, Pal et al. have recently reported^[^ ^36^ ^]^ on an ALA‐based adhesive that can bond various substrates. However, the reported adhesive relies on cation‐containing solvents or heating for polymerization, while the recycling process requires addition of NaOH‐containing solvent.

Using ALA‐derivatives' potential, we introduce a novel solvent‐free, radiation‐curable thermoset adhesive that overcomes these limitations. Our proposed adhesive photocures rapidly (30 s) to bond diverse types of substrates under irradiation at various visible‐light wavelengths (400–650 nm) and enables efficient recycling by a simple household microwave oven, eliminating the need for adding solvents or high temperature processing. This sustainable solution paves the way for adhesive technologies that align with global sustainability goals and industrial needs.

## 2 Results and Discussion

### 2.1 Synthesis and Characterization

**Figure 1:**
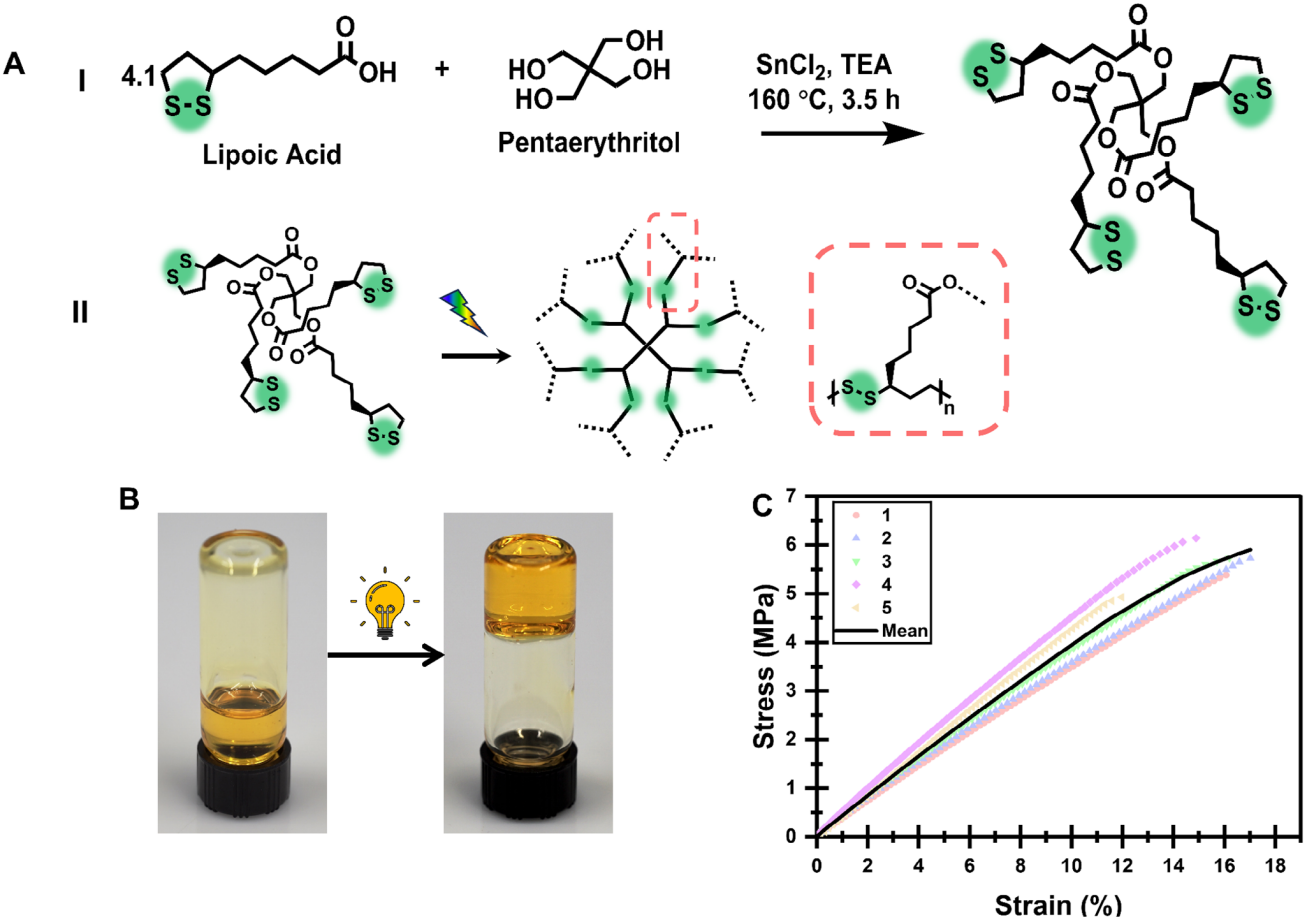
Monomer synthesis and bulk characterization. A) Schematic illustrations of the monomer synthesis (I) (Figure S1, Supporting Information) and the curing process (II). B) A photograph represents the curing process of TetraALA. On the left, the monomer in its liquid form flows to the bottom of the vial. After irradiation for 30 s (when the vial's black lid is on top), the monomer solidifies and remains on the bottom of the vial. C) Tensile tests of the cured monomer after 30 s at 405 nm.

A fully recyclable thermoset adhesive can only be obtained while incorporating fully reversible bonds in the cross‐links or backbone. This study has focused on exploring the potential of cyclic disulfides, specifically α‐Lipoic Acid (ALA), a naturally occurring carboxylic acid that contains a cyclic disulfide functional group. However, more than one functional group is required to achieve a cross‐linked thermoset‐like structure. To address this, ALA was esterified by reacting it with pentaerythritol. A simple “one‐pot” synthesis method was used (**Figure** **1**A; Figure S1A, Supporting Information), which involves mixing the two reagents along with tin (II) chloride (SnCl_2_) as a catalyst,^[^ ^50^, ^51^, ^52^ ^]^ triethylamine (TEA) as a weak base and co‐catalyst, acetone as a solvent, and 1,4‐dioxane as a co‐solvent. Since water, acetone, TEA, and 1,4‐dioxane all have boiling points below 160 °C, they are easily removed upon completion of the reaction, which is performed in an open flux. ^1^H‐NMR (Figure S1B, Supporting Information) and ATR‐IR (Figure S1C, Supporting Information) evaluation of the resulting monomer (**TetraALA**) indicate a complete conversion of the pentaerythritol's hydroxyl groups. However, as an excess of ALA was used, a small amount of non‐esterified ALA remained in the form of ionized carboxylates after reacting with TEA. Furthermore, the process, characterized as a “one‐pot” single‐step synthesis utilizing only commercially available and relatively cost‐effective raw materials (Table S1, Supporting Information), alongside the absence of purification requirements, holds industrial potential regarding viability and scalability.

To evaluate the curing rate of the adhesive, the sample was subjected to irradiation at 405 nm for different periods, resulting in 92.7 ± 2.7% conversion after 30 s (Figure S2, Supporting Information). As shown in Figure 1B, the monomer is a liquid that flows to the bottom of the vial, and after irradiation (when the vial's black lid is on top), the monomer solidifies and remains on the bottom of the vial, forming a yellow brittle solid. Once the photocuring of the composition was established, the cured polymer's glass transition temperature (T_g_) was analyzed through dynamic mechanical analysis (DMA, Figure S3, Supporting Information). The T_g_ was found to be 37 °C, meaning that unlike many reported disulfide‐containing thermosets, this polymer is in its glassy state at room temperature, correlated with its brittleness. Due to the lack of solvents in the composition, the tensile strength of the cured bulk polymer was very high, 5.6 ± 0.5 MPa (Figure 1C), stronger than any other reported ALA‐based polymers.^[^ ^8^, ^36^, ^45^, ^53^ ^]^

### 2.2 Adhesion Evaluation

**Figure 2:**
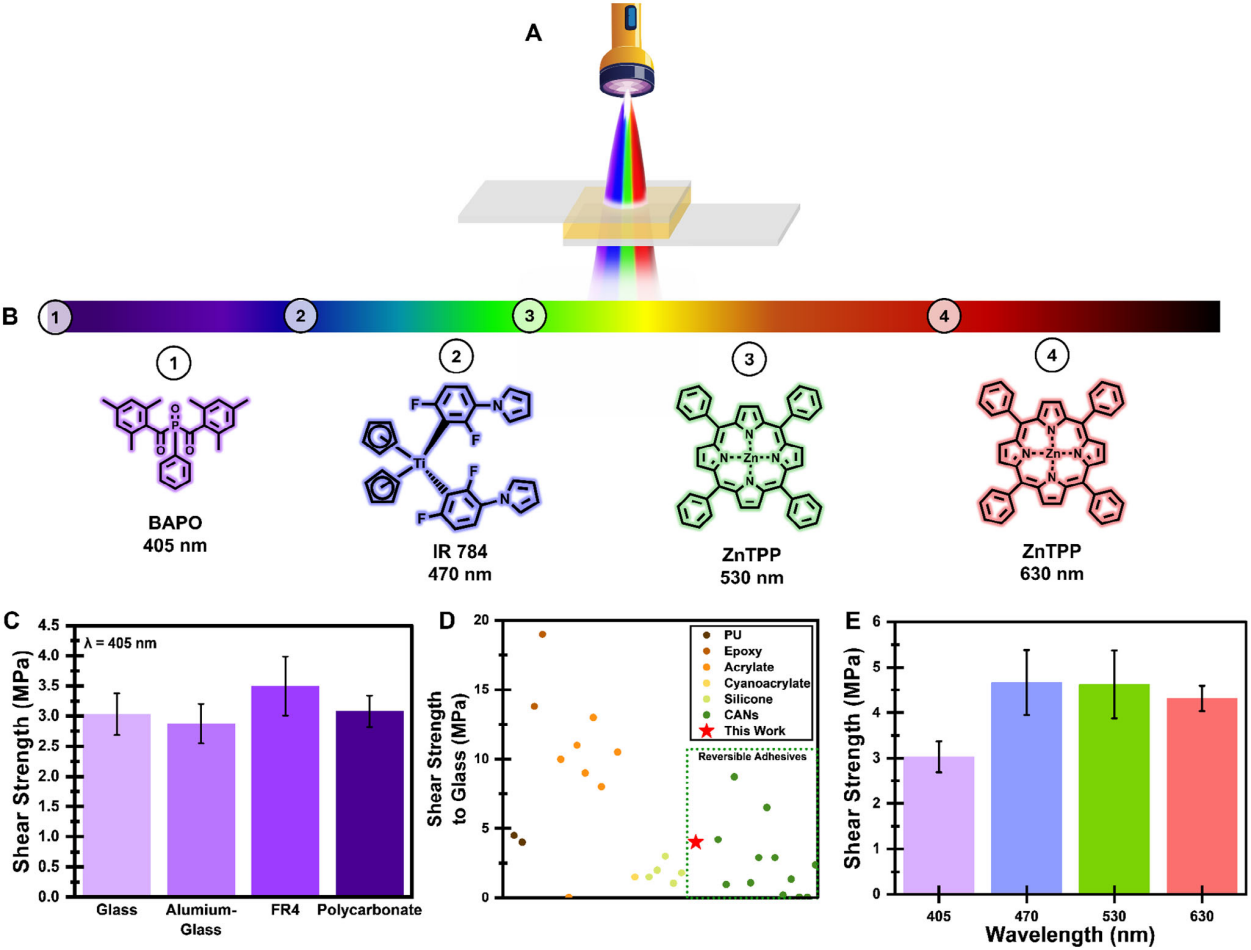
Adhesion analysis. A) Schematic illustration of the adhesion system, including an adhesive layer placed between two substrates, cured by irradiation. B) The visible spectrum and the four photoinitiators that were used in this study (from 1 to 4): Phenylbis(2,4,6‐trimethylbenzoyl)phosphine oxide (**BAPO**, also referred to as Irgacure 819), Bis(.eta.5‐2,4‐cylcopentadien‐1‐yl)‐bis(2,6‐difluoro‐3‐(1H‐pyrrol‐1‐yl)phenyl) titanium (**IR 784**), Zinc (II) tetraphenylporphyrin (**ZnTPP**, both for 3 and 4). C) Lap shear results for different substrates irradiated with BAPO under 405 nm for 30 s. D) Shear strength to glass of **TetraALA** compared to commercial non‐reversible adhesives and literature‐reported reversible adhesives (all raw data is available at Table S3, Supporting Information). E) Lap shear results on treated glass substrates under different wavelengths. All specimens were irradiated for 30 s. All lap shear tests presented in this figure were pre‐treated by mercapto‐silane. All raw data for the lap shear tests are available in Table S2 (Supporting Information).

The adhesion performance of **TetraALA** was evaluated using lap shear tests (a schematic illustration of irradiating the two substrates at the test configuration is shown in **Figure** **2**A). As typically used in the adhesives industry, prior to the adhesion process, a primer was applied to the tested substrates to ensure covalent bonding at the interface. In this case, (3‐Mercaptopropyl)trimethoxysilane, a commonly used silane primer for epoxy resins, was selected due to its commercial availability and because it had been previously reported in thiol‐ALA reactions^[^ ^54^ ^]^ (Figure S4, Supporting Information). This specific primer also ensures that the adhesive will bond to the primer with reversible bonds rather than traditional non‐reversible covalent bonding.

Following the primer treatment, a layer of pre‐cured monomer, at a thickness of 180–200 µm, was applied to one of the substrates. The two substrates were pressed together and irradiated for 30 s. Initially, four substrates were tested: glass, which is commonly used for optical applications; FR4, which is a common substrate in the printed circuit boards industry;^[^ ^55^ ^]^ aluminium, as a representative of metals, and polycarbonate (PC), a transparent, strong amorphous polymer.^[^ ^56^ ^]^ Due to aluminum being non‐transparent for all tested wavelengths (Figure S5, Supporting Information), the adhesion strength to this substrate was conducted on specimens consisting of glass slides and aluminum. Figure 2C shows that almost similar strength was observed for all substrates irradiated under 405 nm. It should be noted that the glass substrates broke under the test conditions; thus, the real adhesion force to the glass was very high and could not be measured. All other substrates showed cohesion failure (Figure S7, Supporting Information). The similarities between the substrates are probably a result of pre‐treatment, which forms a thiol layer on the surface. Moreover, as demonstrated in Table S3 (Supporting Information), the measured adhesion strength to glass is in line with commercial non‐reversible polyurethane adhesives and higher than most literature reported CANs‐based\recyclable adhesives described in the literature (Figure 2D; Table S3, Supporting Information).

One of the drawbacks of ALA‐based adhesives is their dependency on irradiation at a narrow range of light spectrum.^[^ ^33^, ^37^, ^38^, ^39^, ^40^, ^41^, ^42^, ^43^ ^]^ Thus, this study used four visible‐light wavelengths to cure **TetraALA**: 405, 470, 530, and 630 nm. Achieving curing under different wavelengths requires some modifications to the adhesive composition. Based on their light absorbance spectra (Figure S5, Supporting Information), BAPO (Figure 2B1) was chosen for 405 nm, IR 784 (Figure 2B2) was chosen for 470 nm, and ZnTPP (Figure 2B3‐4) was selected for both 530 and 630 nm. Unlike the first two, ZnTPP is a Norrish type II photoinitiator and requires triethanolamine (TEOA) and diphenyl iodonium (Ph2I^+^) salt as proton donors and acceptors.^[^ ^57^ ^]^ Lap shear tests (Figure 2E) were performed for all four wavelengths on glass substrates, resulting in somewhat similar strength (>4 MPa) with a slightly lower value for 405 nm. Initially, this difference was hypothesized to result from different curing penetration depths. However, the penetration depth only at 470 nm was significantly larger (Figure S6, Supporting Information). Thus, as will be discussed later for underwater specimens (see supplementary text and Figure S13, Supporting Information), and as demonstrated for previously reported ALA‐based adhesives,^[^ ^36^ ^]^ it is more likely a result of coordination bonds between ALA's carboxylate or esters groups and the multi‐valence metal ions that reinforced the adhesive.

### 2.3 Recycling

**Figure 3:**
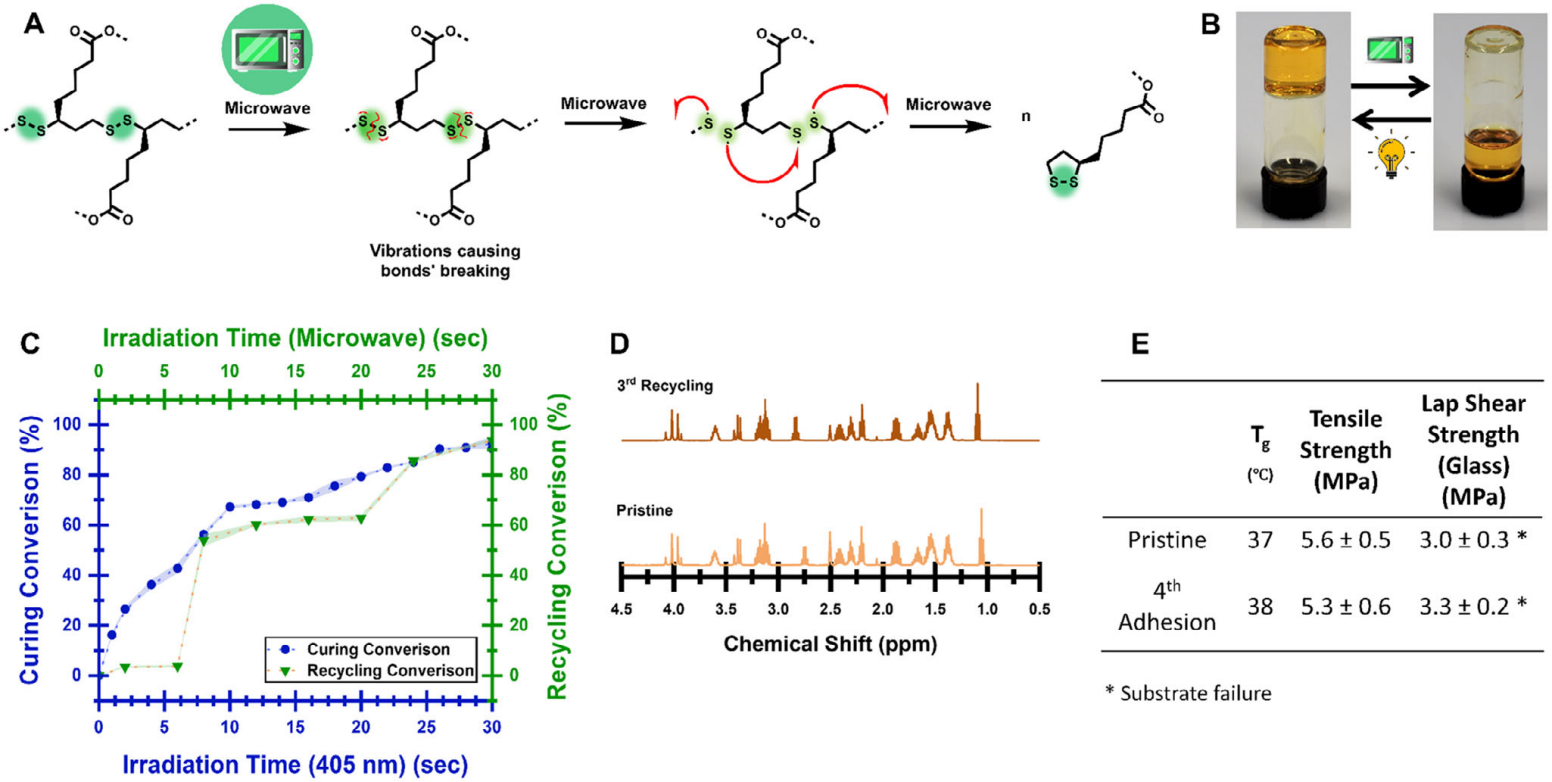
Recycling of TetraALA. A) A schematic illustration of the recycling process. B) a cured polymer (left) transformed back into liquid (right) after 30 s in a 7 W microwave irradiation. C) Photocuring conversion (**405 nm, blue**) and recycling conversion (**microwave,** **green**) as a function of irradiation time. D) ^1^H‐NMR of pristine (**pale orange**) sample and after the 3^rd^ recycling cycle (**dark orange**). The full spectrum is available in Figure S9 (Supporting Information). E) Properties comparison of pristine and 4th cured samples.

Most CANs‐based adhesives reported in the literature rely until now on one (or more) of the following stimuli for recycling: high temperatures,^[^ ^9^, ^10^, ^11^, ^12^ ^]^ adding solvents,^[^ ^58^, ^59^, ^60^ ^]^ or deep UV (200–280 nm) irradiation.^[^ ^22^, ^24^, ^25^, ^26^ ^]^ Specifically, ALA‐based polymers are recycled by heating to >150 °C,^[^ ^61^ ^]^ in a solution with reducing agents,^[^ ^42^, ^54^ ^]^ or in highly basic solvents.^[^ ^62^, ^63^ ^]^ Trying to avoid the need for solvents or elevated temperatures, this study has addressed the recycling challenge through a different approach; based on previous studies by our group,^[^ ^64^ ^]^ it was hypothesized that molecular vibrations resulting from microwave irradiations would cause dissociation of the disulfide bonds to promote depolymerization (**Figure** **3**A,B). Therefore, cured samples were placed in a microwave oven at low intensity to avoid heating for various durations. ATR‐IR measurements revealed that 93.7 ± 0.5% of the polymer was converted to the monomer after only 30 s (Figure 3C; Figure S8, Supporting Information), demonstrating the efficiency of the microwave recycling approach. This was also substantiated by ^1^H‐NMR, even after several recycling cycles, as discussed below (Figure 3D; Figure S9, Supporting Information). To confirm that these results are not due to heating during the microwave irradiation, thermal images (Figure S8, Supporting Information) of samples before and after recycling were taken using a thermal camera, showing the samples’ temperature increased only to ≈47 °C, which is too low to cause any thermal dissociation.

Although a direct assessment of the microwave effect on the molecules cannot be performed inside the microwave oven, the changes observed within the IR spectrum after varying irradiation periods may clarify the underlying mechanism. As illustrated in Figure S8 (Supporting Information), the IR spectrum provides no evidence of thiol group formation, effectively ruling out the possibility of reduction being involved. Nonetheless, some alterations are detected in the C─S vibrations (≈930 cm^−1^). These changes may suggest the proposed mechanism involving the vibrations of the S─S bonds and the linked C─S bonds during microwave irradiation (Figure 3A). Such vibrations destabilize the chemical structure, leading to ring‐closing depolymerization and the reformation of the liquid monomeric form, as demonstrated by the IR and NMR spectra. In addition to the points mentioned above, the involvement of the photoinitiators in the recycling process cannot be completely ruled out, despite the lack of spectroscopic evidence supporting their effect. Nonetheless, because the initiators are essential for the curing process, it would have been impossible to prepare a comparable control system without them. Furthermore, it was postulated by Wanasinghe et al.^[^ ^65^ ^]^ and Huang et al.,^[^ ^66^ ^]^ that lowering bond energies can make dynamic bonds more reversible under mild conditions, mainly if the bonds’ exchanges occur through a dissociative mechanism. Thus, vibrational energy could dissociate bonds by lowering bond exchange energy, resulting in recycling, bonds‐cleavage, or self‐healing.

Specifically, disulfide bonds were shown to react with microwave irradiation, leading to their cleavage. For example, Oh et al. demonstrated^[^ ^67^ ^]^ that microwave irradiation can cleave ALA's disulfide bonds, although they used it for polymerization and surface treatments rather than recycling. Singh et al. also studied the effect of microwave irradiation on disulfide bonds, presenting the use of microwave irradiation to induce self‐healing in disulfide‐containing polymers.^[^ ^68^ ^]^ Their study showed that microwave irradiation can induce cleavage and reformulation of disulfide bonds, thus resulting in self‐healing in their case.

By repeating the photocuring‐depolymerization for several cycles, it was found that **TetraALA** can be recycled and reused up to four times before adding more photoinitiators is required, which is in line with previously reported recyclable adhesives.^[^ ^9^, ^22^, ^25^, ^36^ ^]^ The requirement for the addition of photoinitiators after four cycles is due to the decomposition and depletion of the Norrish Type I photoinitiators (BAPO, IR 784) or the photobleaching of the Type II (ZnTPP) during the photocuring process. It should be noted that the latter is evident, since a color change is observed upon light irradiation. Attempting to cure the samples without adding photoinitiators resulted in a very slow and partial curing. To understand the recycling process's effect on the chemical composition, ^1^H‐NMR and IR evaluation of a pristine sample and a sample after the third recycling was conducted (Figure 3D; Figure S9, Supporting Information), showing no significant changes, proving the reforming of the monomer. Then, the 3^rd^ recycled samples were tested after another (4^th^) curing cycle and were compared with the pristine bulk properties. As shown in Figure 3E, no significant changes were observed either. These similarities between the pristine and the 4‐times cured material are also manifested in the adhesion strength: when testing it on glass, the adhesion strength remained without significant changes, emphasizing the recyclability of **TetraALA** using the microwave.

Further evaluations were conducted as the only observed changes were noted in the bulk elongation (Figure S10, Supporting Information), which increased after recycling. First, a swell test with an ethanol‐acetone mixture was conducted (Figure S11, Supporting Information), showing small changes within the swell ratios (SR) and the gel‐content (GC). It was found that the cross‐linking density was slightly reduced after recycling, resulting in an increase in the SR and a decrease in the GC of ≈35%. As no evidence for hydrolysis or disulfide reduction was found in IR and NMR spectroscopies, these changes are more likely a result of some other side reactions, like disulfide exchanges, which may have occurred during the recycling process. These reactions may cause internal re‐configurations in the cross‐links, in a way that may reduce the effective cross‐links. However, as the amount of disulfide did not change, these changes did not affect the adhesion strength or the bulk mechanical properties. Some evidence for these changes may be found in the samples' creep compliance and stress relaxation (Figure S12, Supporting Information). The pristine sample experienced lower creep compliance and lower stress relaxation than the 4^th^ recycled one, pointing to higher cross‐linking density.

### 2.4 Potential Applications

**Figure 4:**
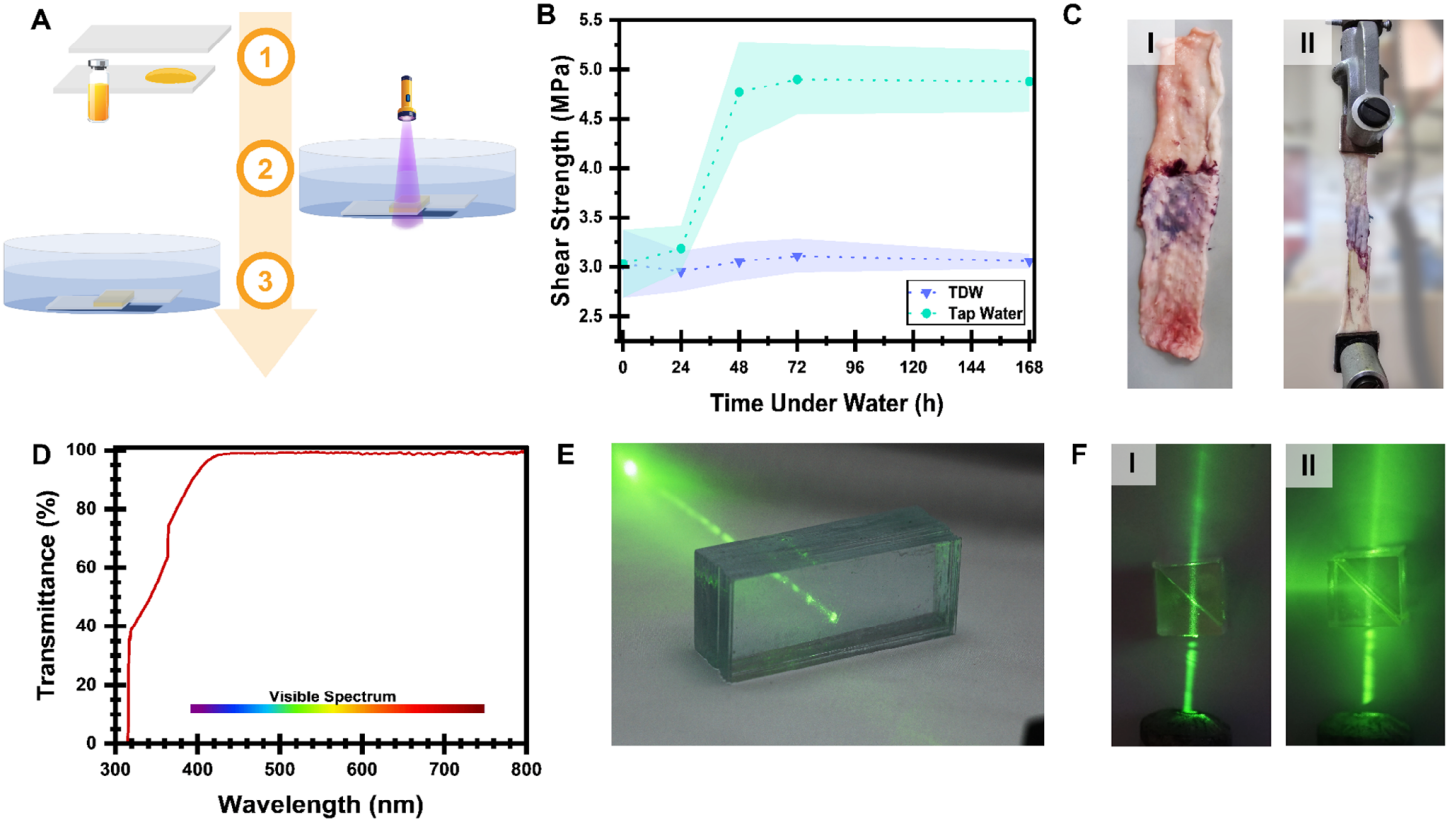
TetraALA potential applications. A) A schematic illustration of the water‐effect analysis. 1, the adhesive is applied on one of the substrates. 2, the two substrates are forced together inside a water bath, forming a thin adhesive layer between them while irradiating under 405 nm. 3, the cured samples are left in water for several hours∖ days. B) Water (**TDW** and **tap water**) effect on adhesion force to glass under irradiation of 405 nm: C) An example of chicken skin lap shear specimen and its test. D) The light transmittance of **TetraALA** thin film (200 µm). E) 22 glass slides that adhere to each other show the transparency of **TetraALA**, as the green laser (535 nm) does not scatter. F) A beam splitter device – two silicone triangular prisms pressed together without (I) and with **TetraALA** (II).

An essential feature of a good adhesive is its ability to function not only in air but also in humid environments, including underwater bonding. First, **TetraALA** was used to bond two glass substrates while being immersed in water. Lap shear specimens were formed from glass substrates and cured in tri‐distilled water (TDW) for 30 s at 405 nm. Then, the specimens were left for 24, 48, 72, and 168 h, for evaluating the water effect on adhesion over time (**Figure** **4**A). As shown in Figure 4B, the adhesion strength was similar to that obtained in air, and the immersion in water for a prolonged duration had no effect. It should be noted that due to the interference of the glass slides with the IR spectrum, as the adhesive itself forms only a ≈200 µm film between the two slides, a direct measurement of the conversion could not be performed. Nonetheless, since the resulting mechanical under dry conditions are similar to that in underwater experiments, it can be assumed that the conversion is similar.

Further evaluation was made using tap water, following the same procedure. Surprisingly, the tap water did not decrease the adhesion force, but increased it by ≈ 60%, reaching an equilibrium at 48 h (Figure 4B). Following the differences in the adhesion strength observed after using the organo‐metallic complexes IR 784 and ZnTPP compared to the organic BAPO,^[^ ^36^ ^]^ it was hypothesized that high‐valence ions that are present in the tap water replaced the ionized TEA, further strengthening the inter and intra‐molecular bonds and thus the adhesive strength. It should be noted that after soaking the sample for 48 and 72 h, only 2.8 ± 0.5% and 3.3 ± 0.1% swelling was observed, respectively (Table C in Figure S13, Supporting Information). These small swelling levels are in line with the relative hydrophobicity of **TetraALA**; yet they indicate that the multivalent ions in the tap water can penetrate the adhesive layer. Furthermore, ATR‐IR spectral analysis (Figure S13, Supporting Information) showed notable changes in the carboxylates’ vibrations and several key regions after water exposure, indicating ion exchange between single‐valence TEA and multi‐valent tap water ions, such as calcium, affecting the material's bonding and properties. These observations are supported by the studies of Pal et al.^[^ ^36^ ^]^ and Fan et al.^[^ ^9^ ^]^ which demonstrated that introducing high valence ions, such as calcium and iron, reinforces lipoic‐based materials.

After observing the good results in water, the adhesive was also tested on chicken skin, representing wet tissues (Figure 4C; Table S2, Supporting Information). The skin's partial transparency to visible light necessitated using the adhesive for 630 nm irradiation, which was irradiated for 1 min. Applying the adhesive proved challenging, due to the oily nature of the skin tissue leading to some incompatibility issues. Despite this, the adhesive's strength was 144.7 ± 17.2 kPa, a superior shear strength compared to other lipoic acid‐based adhesives.^[^ ^36^ ^]^ The achieved strength, which is within the range reported for commercial cyanoacrylate‐based adhesives,^[^ ^69^, ^70^ ^]^ opens the door to future biomedical applications. Nonetheless, as it is outside this study's scope, a further evaluation of biocompatibility of this system is required while taking into consideration the specific requirements of the intended biomedical application.

Taking advantage of the transparency (Figure 4D) and the high refractive index (1.62) of **TetraALA,** it was also assessed as a potential adhesive for optical applications. First, it was tested for optical devices requiring high transparency so that no light scattering would occur. Thus, 22 glass slides were adhered together with a 200 µm adhesive layer between each glass. After curing for 30 s at 405 nm, no scattering of a visible light laser (532 nm) was observed (Figure 4E). As the light penetrates the glass, some reduction in its intensity was observed. Thus, a comparison of 22 glass slides with and without the adhesives’ layers was evaluated. Surprisingly, compared to the non‐adhered glass slides, the measured intensity of the light beam after penetrating the adhesive‐containing slides exhibited a seven times lower reduction in the light intensity due to lower light reflection, emphasizing the potential of TetraALA for such optical applications. The second test was using the adhesive layer for making a beam splitter. Beam splitters are devices that split a light beam into transmitted and reflected beams, usually with 90 ° splitting.^[^ ^71^ ^]^ These devices are key factors in many applications, such as optical fibers and interferometers. The most common structure for such devices is two right‐angled triangular prisms, forming a cube with a thin adhesive layer between them. The two prisms were made from transparent silicone (SYLGARD™ 184). As shown in Figure 4F, a 532 nm laser beam is splitted into two distinctive beams with 90 ° between them, opening the door for further investigations of this adhesive's optical properties and application in optics industries.

## 3 Conclusion

The adhesives industry mainly comprises thermosets as raw materials, resulting in economic shortcomings and environmental challenges. This study presents a fully recyclable, solvent‐free, visible‐light‐curable adhesive based on an α‐lipoic acid derivative. The proposed adhesive cures rapidly under a wide spectral range of visible light, achieving strong and constant adhesion to various substrates, even underwater. Recycling is performed using a low‐intensity house microwave while avoiding the typical need in reversible adhesives for adding solvents or recycling at elevated temperatures, while maintaining the adhesion performance following multiple adhesion‐debonding cycles.

By combining rapid curing, mechanical robustness, and closed‐loop recyclability, this adhesive exemplifies a sustainable approach to thermosets‐based adhesives. Its compatibility with biomaterials, optical systems, and underwater applications highlights its potential for further advancements in adhesive design and material sustainability.

Future studies should elaborate on the microwave‐induced recycling mechanism, employing computational chemistry and other complementary measurements. In view of industrial application of microwave recycling, the proposed approach presents various challenges that should be addressed in the future, for example, suitability to a variety of substrates and to non‐planner substrates, and the optimal ways for recovering the recycled adhesive from the bonded laminates.

## 4 Experimental Section

### Materials

Pentaerythritol (98+%), zinc meso‐tetraphenylprophine (ZnTPP), triethylamine (TEA), and Diphenyliodonium hexafluorophosphate (Ph_2_I^+^) were supplied by Tzamal D‐Chem, Israel. α‐Lipoic Acid (ALA) was purchased from Aaron Chemicals, USA. Tin (II) chloride anhydrous (SnCl_2_, 99%) and 1,4‐dioxane were supplied by Rhenium, Israel. (3‐mercaptopropyl)trimethoxysilane was supplied by Gelest through Tzamal D‐Chem, Israel. Phenylbis(2,4,6‐trimethylbenzoyl)phosphine oxide (BAPO, also referred to as Irgacure 819) and Bis(.eta.5‐2,4‐cylcopentadien‐1‐yl)‐bis(2,6‐difluoro‐3‐(1H‐pyrrol‐1‐yl)phenyl) titanium (Irgacure 784, IR 784), were supplied by IGM Resins, the Netherlands. Ethanol (technical grade, 96%) and acetone (technical grade) were purchased from Bio‐Lab, Israel. Triethanolamine (TOHA) was supplied by Sigma‐Aldrich, Israel. was purchased from Dow Chemicals. All chemicals were used without further purification.

FR4 (G10 epoxy glass laminates) and aluminum (Al) substrates (2.5 cm × 10.1 cm × 2 mm Width × Length × Thickness) were supplied by Arch Materials Services. Polycarbonate (PC) substrates (2.5 cm × 10.1 cm × 4 mm W × L × T) were purchased from Dow Chemicals. For glass substrates, standard microscope slides were used. Chicken skin was bought from the local butcher shop and then cut into samples similar in size to the aluminum and FR4 ones. Before been used, the chicken skin samples were washed carefully with water and dish soap.

### Structural Analysis

Chemicals’ compositions were analyzed using IR and NMR spectroscopy. IR spectroscopy was recorded using ATR‐IR method from 400 to 4000 cm^−1^ on Bruker Alpha‐P machine (Brucker, USA). ^1^H‐NMR spectroscopy was performed using 400 Hz spectrometer (Ascend 400 Neo by Brucker, USA) using tetramethylsilane (TMS) as an internal reference and CDCl_3_ as a solvent.

All Absorbance and transmittance spectra were recorded using a UV‐Vis spectrophotometer (UV‐1800, Shimadzu, Japan) from 1000 to 190 nm using ethanol as a solvent (3.75·10^−6^ g ml^−1^) (Figure S5, Supporting Information). Transmittance of cured thin‐filmed (Figure 4D) was recorded using 200 µm film between two poly(ethylene terephthalate) films (75 µm), which were also recorded as the reference during the measurement.

Swell tests (Figure S11, Supporting Information) were conducted to further evaluate the differences between pristine and recycled materials. According to ASTM D2765‐16, the samples were immersed in a 50:50 ethanol and acetone mixture for 48 h at room temperature. After swelling, the samples were filtered using a Büchner funnel with cellulose filter paper and immediately weighed. The wet samples were then dried in a vacuum oven at 30 °C for 14 h and weighed again. Swelling ratio (SR) and gel‐content (GC) were calculated according to Equations (1) and (2).

(1) $$SR\left(\%\right)=\frac{Swelledsample^{\prime }sweight}{Samples^{\prime }originalweight}\cdot 100$$

(2) $$GC\left(\%\right)=\frac{Samples^{\prime }weightafterdrying}{Samples^{\prime }originalweight}\cdot 100$$

Swell test in tri‐distilled and tap water was conducted for 48 and 72 h, using 200 µ inch^2^ square specimens, representing the adhesive layer in lap shear specimens. All samples were weighed before the test, after the swelling, and after drying in a vacuum oven at room temperature for 2 h followed by 48 h drying in a desiccator. All 18 samples were analyzed in ATR‐IR spectroscopy, following the same method mentioned above.

### Curing Analysis

Conversion of the curing process (Figure S2, Supporting Information) was conducted by recording the changes within the IR spectrum over time. Since both the monomer and the cured polymer contain disulfide bonds, the conversion was calculated by evaluating the changes in the ester bonds (C─O) and C─S bonds, as after curing their mobility reduced, resulting in changes within the signals’ integrals at ≈930 cm^−1^. These signals were normalized to the C─H signals’ integrals at ≈2990 cm^−1^. The IR was recorded after 0, 1, and 2–30 s with measurements every 2 s. These tests were performed three times each irradiation time.

The cure depth evaluation (Figure S8, Supporting Information) at the various wavelengths and with the different photoinitiators was conducted by bonding glass slides, with each slide bonded to the next by a 200 µm adhesive layer. The upper slide was then irradiated. The lower slides were systematically removed until reaching the fully cured slides that remained bonded to each other. At this point, the height of the adhesive structure was measured and recorded as the curing depth for that specific wavelength.

### Adhesion

Curing of TetraALA was conducted under four wavelengths: 405 nm (405 LED lamp with 5.13 mW cm^−2^) using 1 wt.% BAPO as a photoinitiator, 470 nm (470 LED flashlight with 3.62 mW cm^−2^) using 1 wt.% IR 784 as a photoinitiator, 530 nm (520‐540 nm LED flashlight with 3.38 mW cm^−2^), and 630 (630 LED flashlight with 3.63 mW cm^−2^), both using 1 wt.% ZnTPP as a Norrish type‐II photoinitiator with 1 wt.% TOHA and 1 wt.% Ph_2_I^+^. LEDs’ light intensities were measured using THORLabs’ P400 Opitcal Power Meter with 500 mW sensor. LEDs’ emission spectra were measured using StellarNet Spectrometer (Figure S14, Supporting Information).

All adhesion tests were performed using lap shear samples made according to ASTM D5868‐01, D3163‐01, or D1002‐10, depending on the substrates. The underwater samples were prepared following the same procedure, though the irradiation took place when the samples were located underwater (TDW or tap water). Then, the samples were left under water for 24, 48, 72, and 168 h, and immediately tested to measure the water effect on the adhesion strength. Further information may be found in the Supporting Information.

### Mechanical and Viscoelasticity Analysis

Tensile tests of the pristine and recycled samples (Figure S10, Supporting Information) were performed on thin‐films dog‐bones‐like specimens following ASTM D638 type V standard. The tensile tests were performed using a Dynamic Mechanical Analysis (DMA) instrument (TA Instruments’ DMA Q800 V21.3 Build 96, TA Instruments, USA, equipped with 18 N load cell) in a controlled force mode (static) at room temperature with ramp force of 1 N min^−1^.

Lap shear tests (Table S2, Supporting Information) were conducted following ASTM D5868, D3163, or D1002, depending on the substrates (Figure S7, Supporting Information), using a mechanical tester (INSTRON 4481, Instron, USA) equipped with a load cell of 500 N for the pre‐treated glass samples and the chicken skin samples and 5 kN for the rest of the samples. All tests were performed at 5 mm min^−1^. All tests were performed up to failure.

Creep analysis (Figure S12, Supporting Information) was performed using a parallel‐plates rheometer (Discovery HR‐1, TA Instruments, USA), applying a constant force of 100 kN for 60 min. Stress relaxation (Figure S12, Supporting Information) was performed using the same instrument, though at a constant strain of 5% for 60 min.

### Thermal Analysis

Transition temperatures were analyzed using a DMA instrument (TA Instruments’ DMA Q800 V21.3 Build 96, TA Instruments, USA, equipped with 18 N load cell) in a tensile mode, heating from −50 to 150 °C at 3 °C min^−1^ with an amplitude displacement of 15 µm and a frequency of 1 Hz (Figures S3 and S9, Supporting Information). Samples’ temperature dissipation during the recycling process was evaluated using a thermal camera (FLIR‐E63900, FLIR, Sweden) (Figure S8, Supporting Information).

### Optical Samples Preparation

The adhesive's visible light transmittance was demonstrated using 22 glass slides bonded with a 200 µm layer of adhesive between each slide. The adhesive layers were irradiated simultaneously under 405 nm for 30 s. The scattering of a laser pointer (532 nm, 5 mW output) was then analyzed as it passed through the glass structure. A beam splitter device was manufactured to demonstrate the adhesive's suitability as an alternative to traditional adhesives for beam splitter devices. First, two right‐angle triangular prisms were constructed from SYLGARD™ 184. The two prisms were then joined using a 120 µm adhesive layer and cured for 30 s under 405 nm. The final beam splitter device size was 1 cm^3^.

### Recycling

**TetraALA**’s recyclability was analyzed using samples irradiated in a silicone mold for 30 s. The cured samples were then placed in glass vials and heated in a microwave oven (Sauter's microwave, 720 W, Sauter, China) for various durations, using 10% of the microwave's maximum power (72 W, 2.45 GHz). No sample was continuously irradiated for more than 10 s to prevent overheating. Furthermore, each 10 s of irradiation was followed by a 10‐s cooling period for extended irradiation periods. Overall, the energy input was equal to 3000 J.

The conversion of the recycled samples (Figure 3; Figure S8, Supporting Information) was calculated using the same method as for the pre‐recycled samples. This method involved evaluating the changes in the signals’ integrals ≈ 930 cm^−1^ normalized to the C─H signals’ integrals at ≈2990 cm^−1^, resulting in a recycling conversion of 93.7 ± 0.5% after 30 s. Since it was observed that after four recycling processes, the addition of a photoinitiator was necessary for curing, this research focused on properties after a maximum of four curing cycles.

## Conflict of Interest

The authors declare no conflict of interest.

## Author Contributions

N.J., H.D., and S.M. performed conceptualization. N.J. performed synthesis and characterization. N.J., M.C., and R.G. performed Sample preparation. N.J., S.M., S.K., and H.D. wrote‐review & edited. H.D. and S.M. performed validation. H.D. and S.M. performed supervision.

## Supporting information



Supporting Information

## Data Availability

The data that support the findings of this study are openly available in [figshare] at 10.6084/m9.figshare.27968343, reference number [0].
