# Supplementary material for: Untying the Knot: A Fully Recyclable, Solvent‐Free, Wide‐Spectral Photocurable Thermoset Adhesive
Source: Adv Mater. 2025 May 16;37(30):2502040. doi: 10.1002/adma.202502040 (PMC12306384; doi:10.1002/adma.202502040)
Supplement: Supplementary file 1 — Supporting Information [file ADMA-37-2502040-s001.docx]

Supporting Information

Untying the Knot: A Fully Recyclable, Solvent-Free, Wide-Spectral Photocurable Thermoset Adhesive

*Natanel Jarach ^1,2^, Michal Cohen ^2^, Rivka Gitt ^2^, Hanna Dodiuk ^1^†*, Samuel Kenig ^1^, Shlomo Magdassi ^2^†**

^1^ Natanel Jarach, Hanna Dodiuk, and Samuel Kenig

The Department of Polymer Materials Engineering, Pernick Faculty of Engineering, Shenkar – Engineering. Design. Art, Raman-Gan, Israel. E-Mail: hannad@shenkar.ac.il

^2^ Natanel Jarach, Michal Cohen, Rivka Gitt, Shlomo Magdassi

Institute of Chemistry and Center for Nanoscience and Nanotechnology, The Hebrew University of Jerusalem, Jerusalem, Israel.

Email: magdassi@mail.huji.ac.il

* Corresponding authors; these authors contributed equally to this work.

**Keywords:** covalent adaptable networks, vitrimers, adhesives, recycling, thermosets

1. **TetraALA’ s Synthesis**

**TetraALA** (**Figure S 1**) was synthesized from pentaerythritol, α-lipoic acid (ALA), and tin (II) chloride (SnCl_2_) as a catalyst. At first, 0.05 mol of pentaerythritol, 0.21 mol of ALA and 0.002 mol of SnCl_2_ were added to an open round flux. Then, 0.02 mol of triethylamine (TEA), 10 ml of acetone and 4 ml of 1,4-dioxane were added. The mixture was stirred with a magnetic stirrer (350 Hz) at 160 ℃ for 3.5 h using a metallic holder and then let to cool down to room temperature. The sample system was covered with aluminum foil during the entire process to avoid light exposure. The resulting yellow viscous liquid was then analyzed using ^1^H-NMR (**Figure S1-B**) and ATR-IR (**Figure S1-C**). ^1^H NMR (400 MHz, CDCl_3_): 4.13 (t, J = 3.96 Hz, 8H), 3.58 (t, J = 7.75 Hz, 8H), 3.15 (m, 8H), 2.47 (m, 4H), 2.35 (m, 8H), 1.92 (m, 8H), 1.68 (m, 8H), 1.35 (m, 8H). As the used lipoic acid is not 100% pure and has some different isomers, the splitting does not entirely fit the theoretical prediction, resulting in more multiplets. Moreover, a small amount of residual ionized TEA was observed, affecting the integration of the signals. A 100 % conversion of the Pentaerythritol can be concluded as no residual hydroxyl groups were observed. However, since ALA was added in small excess, some free ionized lipoic carboxylates still exist, as shown in the IR spectrum at 1548 cm^-1^. IR (ATR-IR, cm^-1^): 2928, 2854 (C-H stretching), 1729 (C=O stretching), 1548 (residual COO^-^ stretching from free ionized lipoic), 1425 (C-H bending), 1243 (C-O stretching), 1170 (C-O-C stretching), 1045 (C-O-C Stretching), 890 (C-H out-of-plane bending), 740 (C-H out-of-plane bending). As no OH was observed, it can be concluded that all the Pentaerythritol reacted; thus, a 100% conversion can be concluded. UV-Vis Spectroscopy: λ_max_ (nm) = 341, 200.

| **A** | **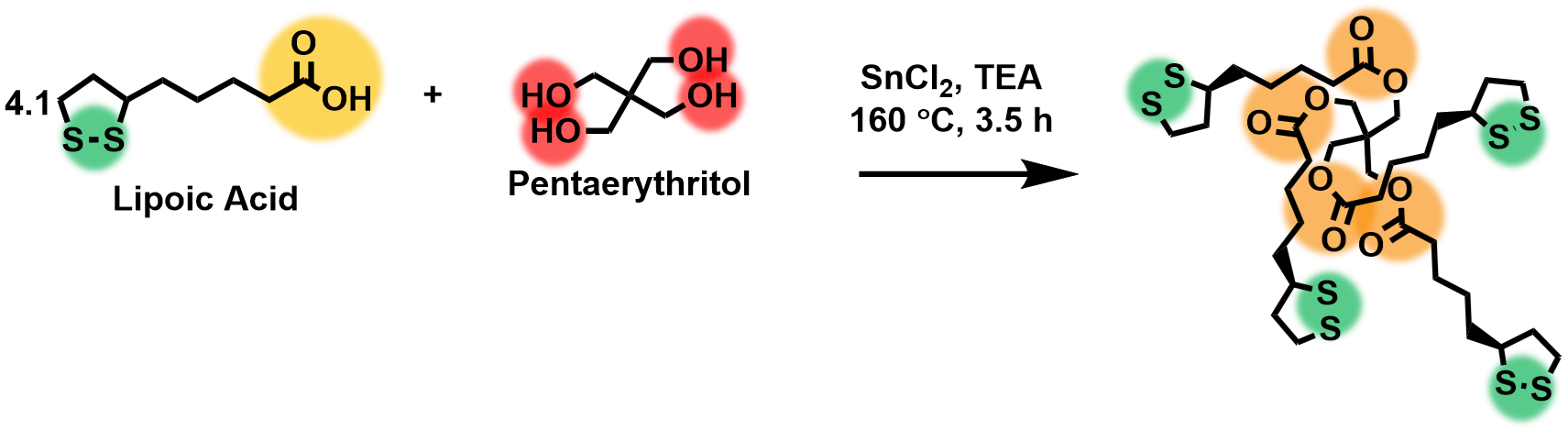** | | |
| --- | --- | --- | --- |
| **B** | **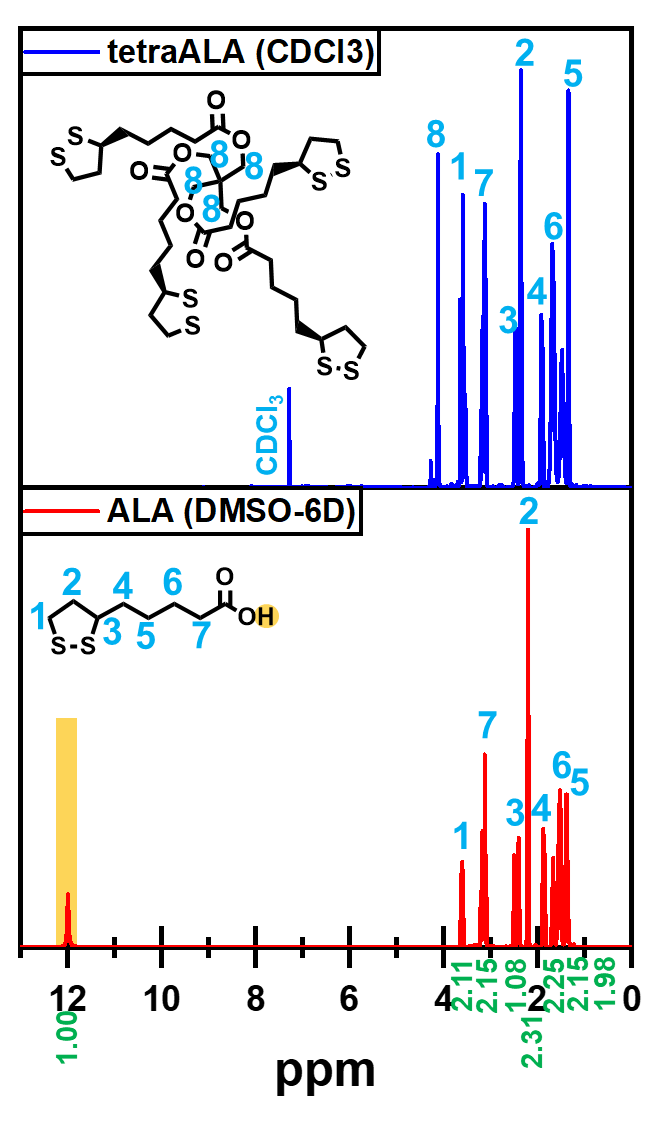** | **C** |  |

**Figure S 1**. **Synthesis of TetraALA**. **A**, a schematic illustration of the monomer synthesis. **B**, ^1^H-NMR spectroscopy at CDCl_3_. **C**, ATR-IR.

**Table S 1.** **Materials’ cost estimations**. Note that cheaper or more expensive prices can be found, depending on the materials’ purity and quality purchased.

| **Material** | **Qty (kg)** | **Unit Price (USD Kg^-1^)** | **Cost**  **(formulation with IR 819)**  **USD Kg^-1^** |  | **Cost**  **(formulation with IR 784)**  **USD Kg^-1^** | **Cost**  **(Type II formulation)**  **USD Kg^-1^** |
| --- | --- | --- | --- | --- | --- | --- |
| **α-Lipoic Acid (ALA)** | 0.93 | 34.00 | 31.62 |  | 31.62 | 31.62 |
| **Pentaerythritol** | 0.07 | 1.50 | 0.105 |  | 0.105 | 0.105 |
| **Irgacure 819** | 0.01 (only in formulation 819) | 5.00 | 0.05 |  | 0 | 0 |
| **Irgacure 784** | 0.01 (only in formulation 784) | 10.00 | 0 |  | 0.10 | 0 |
| **Diphenyliodonium hexafluorophosphate** | 0.01 (only in formulation II) | 0.10 | 0 |  | 0 | 0.001 |
| **Triethanolamine (TEA)** | 0.01 (only in formulation II) | 1.30 | 0 |  | 0 | 0.013 |
| **Zinc meso-tetraphenylporphyrin (ZnTPP)** | 0.01 (only in formulation II) | 11000 | 0 |  | 0 | 110 |
| ***Total*** | **** **** | **** **** | **31.8** |  | **31.8** | **141.7** |

1. **Bulk Characterization**

The polymer curing conversion was evaluated at different irradiation periods at 405 nm, followed by an evaluation of the changes in IR spectroscopy. The main challenge in this evaluation was that, unlike traditional radiation-cured adhesives, which contain double bonds that react and disappear after curing, **TetraALA** contains disulfides in its monomeric and polymeric forms. However, after the curing termination, the constraints on the ester cross-links increased, resulting in changes within their vibrations, especially in the 930 cm^-1^ regions (C-O, but also C-S) (**Figure S 2**). When normalizing it to the internal C-H bonds (2990 cm^-1^), a 92.7 ± 2.7 % conversion was calculated after 30 s (**Figure S 2**). Hence, this time was chosen as the curing time for the adhesive.

| **A** |  | **B** |  |
| --- | --- | --- | --- |

**Figure S 2. Curing conversion analysis of TetraALA. A**, Conversion (%) Vs. irradiation time. The blue shade represents the standard deviation after three tests for each period. **B**, A representative ATR-IR spectroscopy of all irradiation periods. All tests were conducted using 405 nm LED (5.13 mW cm^-2^).

**Figure S 3**. **Dynamic mechanical analysis** (DMA) results for pristine sample. The test was conducted in tensile mode heating at 3 ℃ min^-1^.

1. **Adhesion**
   1. **Silane Pre-treatment**

At first, a lap-shear test was conducted on non-treated glass slides, resulting in relatively poor lap-shear strength to glass (under 405 nm) compared to the bulk strength, with values of 1.5 ± 0.2 MPa and 5.6 ± 0.5 MPa, respectively (**Figure S 4**). Moreover, the fractured specimens demonstrated significant adhesive failure, characterized by the complete separation of the adhesive from the glass substrate, indicating an incompatibility between the adhesive and the substrate. The incompatibility is also evident in their hydrophilic properties (**Figure S 4**); the glass had a water contact angle of 18.4 ± 0.7 °, while **TetraALA** exhibited a contact angle of 66.3 ± 0.6 °. Therefore, it was concluded that a pre-treatment of the glass substrate is necessary.

To increase the adhesion strength, silane treatment was performed on all tested slides according to the literature and Gelest protocol ^[2]^. 2 wt% of the (3-mercaptopropyl)trimethoxysilane was added to 98 wt% ethanol (technical, 96 % with 4 % water). The pH was adjusted to 4.5-5.5 by adding Triethanolamine (TOHA). The mixture was allowed to mix for 5 min. Then, the substrates were immersed in the mixture for 2 min without mixing, followed by quick dipping in ethanol. The substrates were left to dry in the hood for an hour and then heated to 120 ℃ for 2 h. To improve the silane treatment, FR4 (epoxy\glass fibre composites) and polycarbonate (PC) substrates were first exposed to air plasma treatment for 1 min. The success of the silane treatment was measured using a contact angle test (**Figure S 4**) performed on a contact angle meter (OCA 15 DataPhysics, Germany) using a sessile drop method. Each measurement involved depositing a 5 μL droplet of distilled water onto the surface of the tested substrates before and after the silane treatment. The measurements were performed three times.

| **A** |  | **B** |  |
| --- | --- | --- | --- |

**Figure S 4. Silane Treatment.** **A**, Lap shear strength of glass substrates irradiated at 405 nm for 30 s before (**pale blue**) and after (**dark blue**) the silane treatment. **B**, Changes within the contact angles of different substrates before (pale colors) and after the silane treatment (dark colors): Glass (**blue**), aluminium (**grey**), Polycarbonate (**green**), and FR4 (**orange**). This figure also presents the contact angle of pristine (pale purple) and recycled (4^th^ curing, **dark purple**) bulk polymer.

- 1. **Specimen’s preparations**

Phenylbis(2,4,6-trimethylbenzoyl)phosphine oxide (**BAPO**, also referred to as Irgacure 819) and Bis(.eta.5-2,4-cylcopentadien-1-yl)-bis(2,6-difluoro-3-(1H-pyrrol-1-yl)phenyl) titanium (Irgacure 784, **IR 784**) formulations were prepared as follows: 2 g of the sample, containing 1.98 g of TetraALA and 0.02 g of the photoinitiator, was mixed using vortex. Then, the mixture was ultrasonicated in a bath ultrasonicator (80 Hz, Elmasonic P, Elma Schmidbauer GmbH, Germany) at 70 ℃ for 3 min, followed by another vortex mixture. Then, the mixture was ultrasonicated for six more min until a homogeneous mixture was obtained. zinc meso-tetraphenylprophine (ZnTPP)-containing samples were prepared by first adding 0.02 gr ZnTPP to 1.94 g of TetraALA. The mixture was vortex mixed and then ultrasonicated in a bath ultrasonicator (same one) for 3 min at 70 ℃. The mixture was vortex mixed and then ultrasonicated again under the same conditions for 6 more min. 0.02 g of TEOH and 0.02 g of diphenyliodonium hexafluorophosphate (Ph_2_I^+^) were added, vortex mixed and ultrasonicated again for another 2 min at 70 ℃ until a homogeneous mixture was obtained.

All adhesion tests were performed using lap shear samples made according to ASTM D5868-01, D3163-01, or D1002-10, depending on the substrates. A thin film of the adhesive formulation was applied to one of the substrates. Then, the substrates were pressed together using two metallic clips and irradiated for 30 s under the relevant light source.

| **A** |  | **B** |  |
| --- | --- | --- | --- |

**Figure S 5. UV-NIR Spectra**. **A**, Absorbance spectra of the used photoinitiators: **BAPO** – purple, **IR 784** – Blue, **ZnTPP** – red. **B**, light transmittance of three of the used substrates, excluding aluminium that is non-transparent.

**Figure S 6. Cure Depth analysis**. Glass slides were adhered together, with each slide connected to the next by a 200 µm adhesive layer. The upper slide was then irradiated using **405**, **470**, **530**, and **630** nm wavelengths. The lower slides were removed until reaching the fully cured slides. At this point, the height of the adhesive structure was measured.

- 1. **Lap Shear Strength**

| **A** | 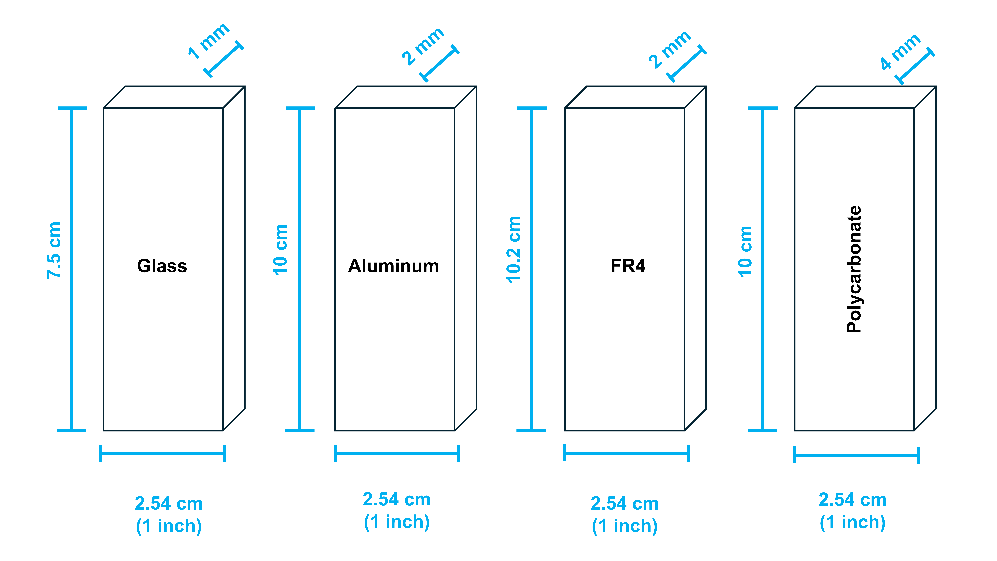 | | **B** | 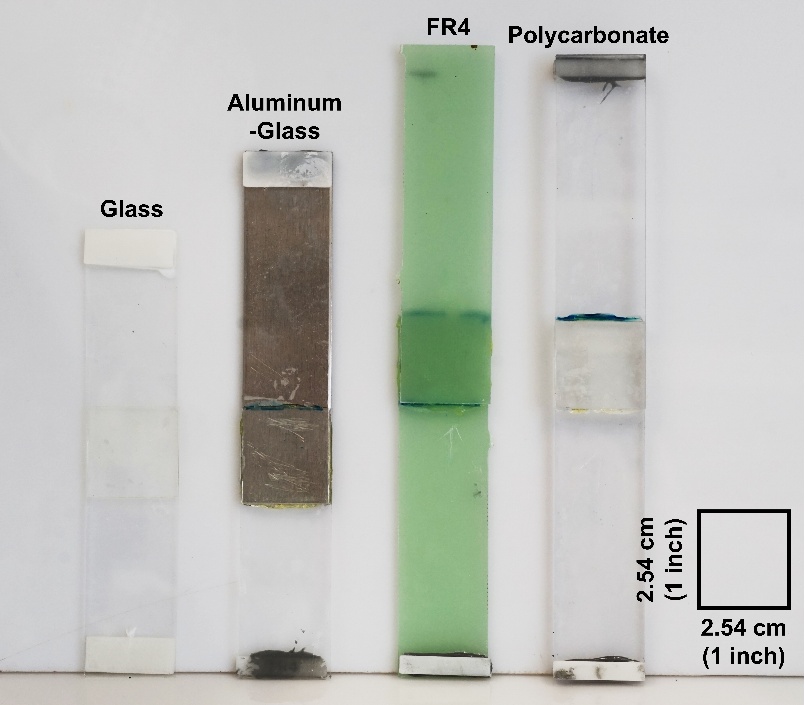 |
| --- | --- | --- | --- | --- |
| **C** | | 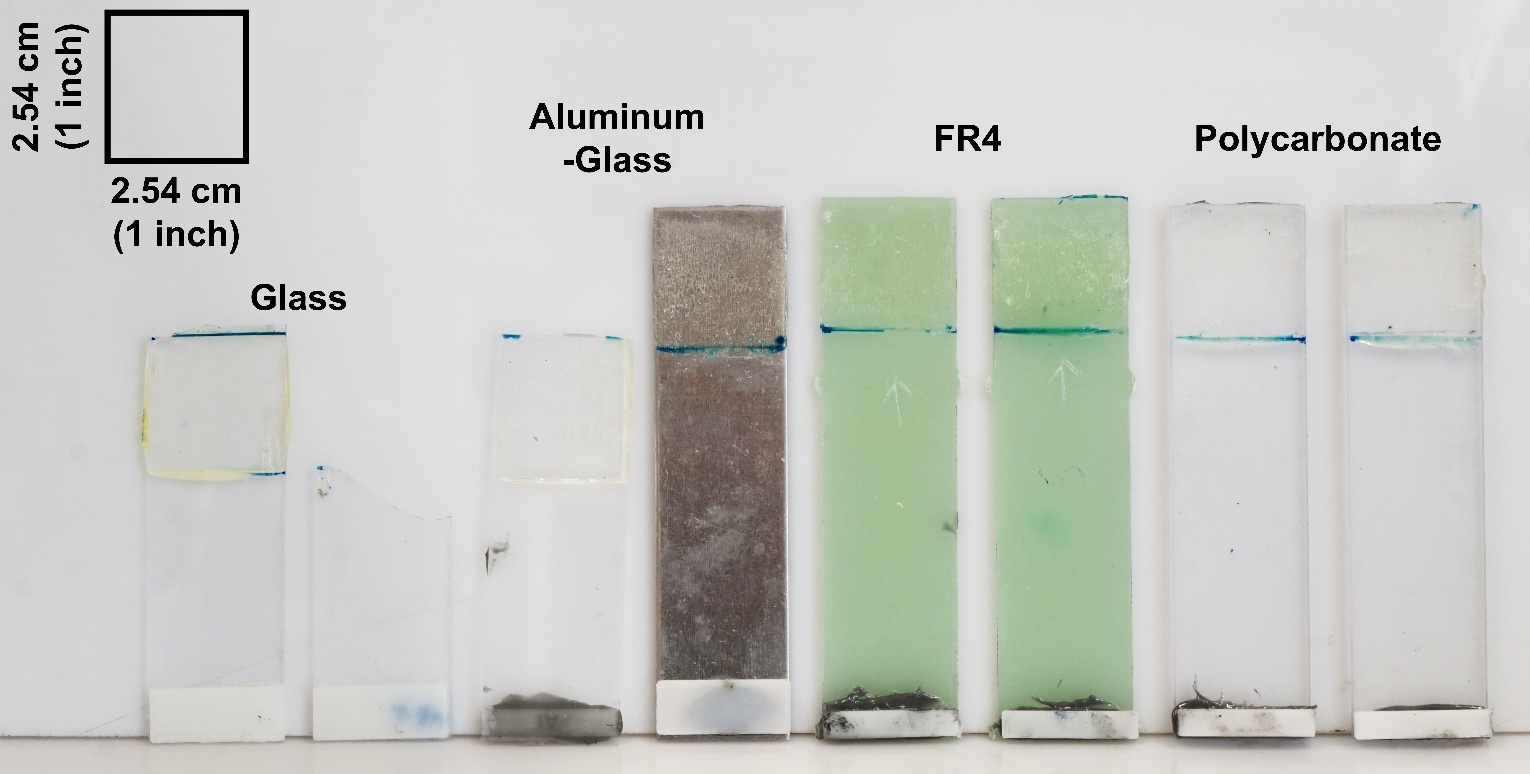 | | |

**Figure S 7**. **Lap-shear specimens.** **A**, The lap-shear specimens’ dimensions. **B**, Lap-shear specimens with BAPO as photoinitiator. The adhesion area is ~6.45 cm^2^ (1 inch^2^), according to the ASTM standards. As shown in the photos, the adhesive forms thin transparent layers that maintain its shape even after several weeks. **C**, Specimens that were irradiated under 405 nm wavelength after failure. All specimens, apart from the glass, underwent cohesive failures. All glass specimens underwent a substrate failure (the adhesion area remained untacked).

**Table S 2. Raw data of all lap shear tests**. All results in MPa except of Chicken skin that is in kPa. The raw data of the tests is available in Figshare. All specimens were tested until a complete failure of either the adhesive (cohesive failure) or the substrate.

| **Sample** | **Untreated Glass** | **Treated Glass** | **Al-Glass** | **FR4** | **Polycarbonate** | **Recycled (4th curing)** | **470 nm** | **530 nm** | **630 nm** | **Dry** | **24h in tap water** | **48h in tap water** | **72h in tap water** | **168h in tap water** | **24h in TDW ^b^** | **48h in TDW ^b^** | **72h in TDW ^b^** | **Chicken Skin ^a^** |
| --- | --- | --- | --- | --- | --- | --- | --- | --- | --- | --- | --- | --- | --- | --- | --- | --- | --- | --- |
| 1 | 1.50 | 2.95 | 3.04 | 3.83 | 3.24 | 3.27 | 3.45 | 4.17 | 4.02 | 2.95 | 2.93 | 4.64 | 4.85 | 4.88 | 2.74 | 3.22 | 3.08 | 155.62 |
| 2 | 1.29 | 2.67 | 2.52 | 3.24 | 2.72 | 2.98 | 5.12 | 5.91 | 4.82 | 2.67 | 3.37 | 4.04 | 4.50 | 4.66 | 2.70 | 2.76 | 3.42 | 159.46 |
| 3 | 1.24 | 2.96 | 2.53 | 2.99 | 2.88 | 3.21 | 4.59 | 4.65 | 4.19 | 2.96 | 3.34 | 4.83 | 5.35 | 4.64 | 3.14 | 3.22 | 2.97 | 138.94 |
| 4 | 1.77 | 3.60 | 3.22 | 3.69 | 3.34 | 3.35 | 5.06 | 4.33 | 4.22 | 3.60 | 2.94 | 5.48 | 5.00 | 5.30 | 3.18 | 3.08 | 3.13 | 117.18 |
| 5 | 1.65 | 2.97 | 3.07 | 3.03 | 3.19 | 3.45 | 5.11 | 4.08 | 4.20 | 2.97 | 3.36 | 4.85 | 4.49 | 4.58 | 3.06 | 3.00 | 2.95 | 152.33 |
| 6 |  |  |  | 4.20 |  | 3.50 |  |  | 4.44 |  |  |  | 5.21 | 5.23 | 2.90 |  | 3.12 |  |
| Mean (MPa) ^a^ | **1.5** | **3.0** | **2.9** | **3.5** | **3.1** | **3.3** | **4.7** | **4.6** | **4.3** | **3.0** | **3.2** | **4.8** | **4.9** | **4.9** | **3.0** | **3.1** | **3.1** | **144.7** |
| SD | 0.2 | 0.3 | 0.3 | 0.5 | 0.3 | 0.2 | 0.7 | 0.7 | 0.3 | 0.3 | 0.2 | 0.5 | 0.4 | 0.3 | 0.2 | 0.2 | 0.2 | 17.2 |
| SD (%) | 15.2 | 11.4 | 11.3 | 13.9 | 8.5 | 5.7 | 15.4 | 16.2 | 6.5 | 11.4 | 7.2 | 10.8 | 7.3 | 6.4 | 6.9 | 6.3 | 5.4 | 11.9 |

^a^ Chicken skin results are in kPa.

^b^ TDW – Triple distilled water.

**Table S 3. Lap shear strength on glass substrates** of industrial non-reversible adhesives and literature reversible (CANs) adhesives.

| **Name** | **Family** | **Shear Strength to Glass (MPa)** | **References** |
| --- | --- | --- | --- |
| Sikaflex® 296 | PU ^a^ | 4.5 | ^[3]^ |
| EP62-1LPSP | Epoxy | 13.8 | ^[4]^ |
| Permabond UV620 | Acrylate | 10 | ^[5]^ |
| Loctite Glass Glue | Cyanoacrylate | 1.5 | ^[6]^ |
| Dymax UV Curable Adhesive | Acrylate | 0.01 | ^[7]^ |
| 3M™ 08603/08615/08616 | PU | 4 | ^[8]^ |
| Dowsil EA-3838 | Silicone | 1.5 | ^[9]^ |
| Dowsil EA-2626 | Silicone | 2 | ^[10]^ |
| Quilosa FMS | Silicone | 3 | ^[11]^ |
| Silirub 2 | Silicone | 1.05 | ^[12]^ |
| Plexus™MA425 | Acrylate | 11 | ^[13]^ |
| SikaFast®-5215 | Acrylate | 9 | ^[14]^ |
| PERMABOND® UV610 | Acrylate | 13 | ^[15]^ |
| PERMABOND® UV625 | Acrylate | 8 | ^[16]^ |
| PERMABOND® TA4246 | Acrylate | 30 | ^[17]^ |
| Sikaflex® 221 | Silicone | 1.8 | ^[18]^ |
| Our | This Work | 4 | ^[19]^ |
| Araldite® 2011 | Epoxy | 19 | ^[20]^ |
| Vitralit UV 2415 | Acrylate | 10.5 | ^[20]^ |
| PU-containing Boronate Ester | CANs ^b^ | 4.2 | ^[21]^ |
| Polyurethane containing boronic esters bonds derived from catechol | CANs | 0.96 | ^[22]^ |
| SBO-epoxy (transesterification) | CANs | 8.73 | ^[23]^ |
| Polystyrene-b-poly(ethylene-co-butylene)-b-polystyrene (SEBS) containing boronic esters bonds | CANs | 39.6 | ^[24]^ |
| 1,2,3-triazolium-containing polymers | CANs | 1.08 | ^[25]^ |
| Polymer containing disulfide bonds | CANs | 2.9 | ^[26]^ |
| Polymer containing disulfide bonds | CANs | 6.5 | ^[27]^ |
| Polythiourethane containing thiocarbamate | CANs | 2.9 | ^[28]^ |
| Siloxane oligomers functionalized with azobenzene groups | CANs | 0.21 | ^[29]^ |
| Spiropyran homopolymers based materials | CANs | 1.33 | ^[30]^ |
| Acrylic-Urushiol-boronic acid | CANs | 0.05 | ^[31]^ |
| Alginate-boronic acid hydrogel | CANs | 0.032 | ^[32]^ |
| Anthracene-based acrylate polymer | CANs | 2.37 | ^[33]^ |

^a^ PU – polyurethane. ^b^ CANs – covalent adaptable networks adhesives. Also known as debonding on demand adhesives, vitrimeric adhesives, and recyclable adhesives.

1. **Recycling**
   1. **Recycling Analysis**

| **A** |  | | **B** |  | |
| --- | --- | --- | --- | --- | --- |
| **C** | I | 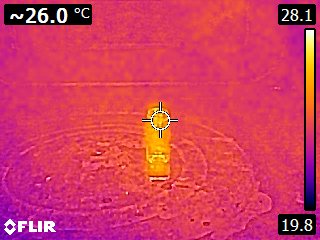 | | II | 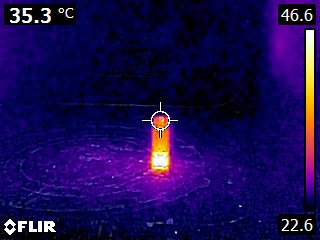 |

**Figure S 8. Recycling conversion analysis of TetraALA and thermal dissipation during the process**. **A**, recycling conversion (%) Vs. irradiation time at the microwave oven (72 W). The red shade represents the standard deviation after three tests for each period. **B**, A representative ATR-IR spectroscopy of all irradiation periods. Other spectra can be found in the online raw data in Figshare. **C**, Thermal photos of the samples before (**I**) and after (**II**) the 30 s recycling process.

- 1. **Comparison of Pristine and Recycled**

| **A** | 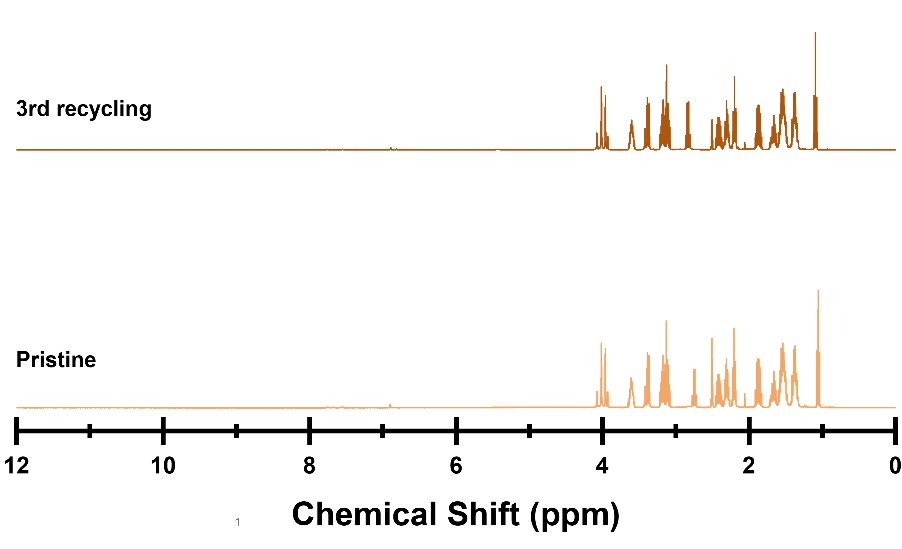 | **B** |  |
| --- | --- | --- | --- |

**Figure S 9**. **Spectroscopy comparison of pristine and recycled samples. A,** Full ^1^H-NMR spectra of **pristine** and **4^th^ cured** samples. **B**, ATR-IR spectra of **pristine** and **4^th^ cured** samples.

| **A** |  | **B** |  |
| --- | --- | --- | --- |
| **C** |  | **D** |  |

**Figure S 10. Bulk comparison of pristine and recycled samples**. Tensile tests of pristin**e** (**A**) and recycled (**B**, 4^th^ curing cycle) specimens. Dynamic mechanical analysis (DMA) of pristine (**C**) and recycled (**D**, 4^th^ curing cycle) samples.

**Figure S 11. Swell tests and gel content** calculations for pristine and recycled (4^th^ curing cycle) samples.

| **A** |  | **B** |  |
| --- | --- | --- | --- |

**Figure S 12. Viscoelastic properties of pristine and recycled samples**. **A,** Creep analysis for 60 min. **B**, stress relaxation. For both tests, **pristine** is presented in pale orange whereas the **recycled sample** is presented in dark orange.

1. **Potential Applications**
   1. **Water Effects Analysis**

The ATR-IR analysis (**Figure S 12**) shows spectral changes before and after the water exposure, strengthening the hypothesis of ion exchange and structural reconfiguration. Specifically, changes within the asymmetric stretching intensities of carboxylate groups (COO^-^) at 1554 cm^-1^ and 1648 cm^-1^ were observed. Changes in the symmetric stretching region were observed within the intensity at 1395 cm^-1^. Furthermore, the C=O signal\ shoulder at 1712 cm^-1^ is absent after the water exposure, and some changes are also observed in the 1655 cm^-1^ signal and within the spectral features in the fingerprint region. These observations can result from the exchange of single-valence ions (ionized TEA) with multi-valence ions, like calcium (Ca^2+^) or magnesium (Mg^2+^), which exist in tap water. This ion exchange process leads to substantial alterations in the bonding environment, with implications for the material's structural and mechanical properties.

| **A** |  | **B** |  |
| --- | --- | --- | --- |
|  |  |  |  |
| **C** | \|  \| Initial (mg) \| Wet (mg) \| Dry (mg) \| **Swell Ratio (%)** \| **Swell Ratio-Av (%)** \| **Swell Ratio-SD** \| **Dry-Vs.-Wet (%)** \| **Dry-Vs.-Wet-Av (%)** \| **Dry-Vs.-Wet-SD** \| \| --- \| --- \| --- \| --- \| --- \| --- \| --- \| --- \| --- \| --- \| \| 48 hours \| 333.9 \| 344.9 \| 333.9 \| 3.29 \| 2.8 \| 0.5 \| 100 \| 99.97 \| 0.02 \| \| 449.2 \| 460.0 \| 449.0 \| 2.40 \| 99.95 \| \| 287.9 \| 295.4 \| 287.8 \| 2.60 \| 99.96 \| \| 72 hours \| 269.1 \| 278.4 \| 269.0 \| 3.46 \| 3.3 \| 0.1 \| 99.96 \| 99.93 \| 0.03 \| \| 202.0 \| 208.5 \| 201.8 \| 3.22 \| 99.90 \| \| 280.8 \| 289.9 \| 280.6 \| 3.24 \| 99.93 \| | | |

**Figure S 13. Water effects’ analysis**. ATR-IR spectra of samples swelled in water for 48 h (**A**) and 72 h (**B**). Three measurements are presented: “**Initial**” – representing the sample before immersing in water, “**Wet**” – a sample that has been taken from water and immediately weighed, and “**Dry**” – a sample weighed after drying in a vacuum oven at room temperature for two hours followed by 48 h drying in a desiccator. These graphs contain the average curves of three samples each. **In orange** – samples before swelling. **In blue** – samples after swelling. **In red** – samples after drying for 48 h under vacuum in a desiccator. **C**, Swelling measurements of the samples in water.

- 1. **Light-Source Emission**

**Figure S 14**. **Emission spectra** of the different LEDs used in this study.

**References**

[1] H. Shy, P. Mackin, A. S. Orvieto, D. Gharbharan, G. R. Peterson, N. Bampos, T. D. Hamilton, *Faraday Discuss* **2014**, *170*, 59.

[2] Gelest, Inc, *Silane Coupling Agents: Connecting Across Boundaries*, **2014**.

[3] “Sikaflex®-296 | Glass Bonding,” can be found under https://industry.sika.com/en/home/marine/leisure-boats-andyachts/glass-bonding/sikaflex-296.html, **n.d.**

[4] “EP62-1LPSP,” can be found under https://www.electronicspecifier.com/products/component-management/epoxy-has-a-tensile-lap-shear-strength-of-more-than-2-000psi, **n.d.**

[5] “PERMABOND UV620 - Samaro,” can be found under https://www.samaro.fr/en/product/permabond-uv620/, **n.d.**

[6] “Loctite Glass Glue,” can be found under https://www.loctiteproducts.com/products/central-pdp.html/loctite-glass-glue/SAP_0201DFL029X5.html, **n.d.**

[7] “MD® 1-CN003 UV/Visible Light-Curable Adhesive for Bonding Metals | Enhanced Reader,” can be found under https://dymax.com/content/download/3580/file_archived/1-CN003%20PDS.pdf, **n.d.**

[8] “3M 08603/08615/08616 One Part Polyurethane Bonding Adhesive,” can be found under https://multimedia.3m.com/mws/media/1809189O/3m-glass-bonding-adhesive-08603-08615-08616.pdf, **n.d.**

[9] “DOWSIL^TM^ EA-3838,” can be found under https://www.biesterfeld.com/en/mx/service/competence-in-solutions/adhesives/, **n.d.**

[10] “DOWSIL^TM^ EA-2626 Adhesive | Dow Inc.,” can be found under https://www.dow.com/en-us/pdp.dowsil-ea-2626-adhesive.04085662z.html#overview, **n.d.**

[11] “Quilosa FMS INSTANT,” can be found under https://www.glueguru.co.nz/site/glueguruunleashed/images/Brands/Quilosa/Technical%20Files/QUILOSA%20FMS%20TDS.pdf, **n.d.**

[12] “Silirub 2 | Soudal,” can be found under https://www.soudal.za.com/pro/products/sealants/silicone-sealants/silirub-2, **n.d.**

[13] “PLEXUS MA425,” can be found under https://www.ulbrich-group.com/chemical-technical-products/TDS_PLEXUS_MA425_eng.pdf, **n.d.**

[14] “Sikafast® 5215,” can be found under https://gbr.sika.com/dms/getdocument.get/d95a6cd7-6d28-3548-a848-ea4ebd7a2318/SikaFast_5215_e_10_03.pdf, **n.d.**

[15] “PERMABOND UV610,” can be found under https://www.permabond.com/wp-content/uploads/2016/04/UV610_TDS.pdf, **n.d.**

[16] “PERMABOND UV625,” can be found under https://www.permabond.com/wp-content/uploads/2016/04/UV625_TDS.pdf, **n.d.**

[17] “TA4246_TDS,” can be found under https://www.permabond.com/wp-content/uploads/2016/04/TA4246_TDS.pdf, **n.d.**

[18] “Sikaflex®-221 | Adhesive Sealants,” can be found under https://industry.sika.com/en/home/transportation/sealants/adhesive-sealants/sikaflex-221.html, **n.d.**

[19] “Araldite 2011,” can be found under https://docs.rs-online.com/7e8f/0900766b80034503.pdf, **n.d.**

[20] “Vitralit UV 2415,” can be found under https://www.panacol.com/panacol/datasheets/vitralit/vitralit-uv2415-english-tds-panacol-adhesive.pdf, **n.d.**

[21] J. Li, C. Hu, B. Yang, Z. Ning, Y. Zeng, *Polymer (Guildf)* **2022**, *256*, 125227.

[22] Y. Yang, F. S. Du, Z. C. Li, *ACS Appl Polym Mater* **2020**, *2*, 5630.

[23] Z. Liu, Z. Song, B. Lv, Z. Qiu, *Polymers 2023, Vol. 15, Page 3488* **2023**, *15*, 3488.

[24] M. A. Rahman, C. Bowland, S. Ge, S. R. Acharya, S. Kim, V. R. Cooper, X. Chelsea Chen, S. Irle, A. P. Sokolov, A. Savara, T. Saito, *Sci Adv* **2021**, *7*, DOI 10.1126/SCIADV.ABK2451.

[25] M. M. Obadia, A. Jourdain, P. Cassagnau, D. Montarnal, E. Drockenmuller, *Adv Funct Mater* **2017**, *27*, 1703258.

[26] B. T. Michal, E. J. Spencer, S. J. Rowan, *ACS Appl Mater Interfaces* **2016**, *8*, 11041.

[27] B. T. Michal, E. J. Spencer, S. J. Rowan, *ACS Appl Mater Interfaces* **2016**, *8*, 11041.

[28] C. Cui, X. Chen, L. Ma, Q. Zhong, Z. Li, A. Mariappan, Q. Zhang, Y. Cheng, G. He, X. Chen, Z. Dong, L. An, Y. Zhang, *ACS Appl Mater Interfaces* **2020**, *12*, 47975.

[29] R. H. Zha, G. Vantomme, J. A. Berrocal, R. Gosens, B. De Waal, S. Meskers, E. W. Meijer, *Adv Funct Mater* **2018**, *28*, 1703952.

[30] K. Imato, K. Momota, N. Kaneda, I. Imae, Y. Ooyama, *Chemistry of Materials* **2022**, *34*, 8289.

[31] H. Tang, Q. Chen, X. Ke, H. Wang, M. Li, J. Xie, J. Luo, J. Li, *Ind Eng Chem Res* **2023**, *62*, 19690.

[32] S. H. Hong, S. Kim, J. P. Park, M. Shin, K. Kim, J. H. Ryu, H. Lee, *Biomacromolecules* **2018**, *19*, 2053.

[33] Y. Feng, X. Han, Y. Ji, S. Li, K. Dong, S. Liang, Y. Ma, Y. Yang, F. Liu, *J Adhes* **2024**, *100*, 200.
